# miR-4651 inhibits cell proliferation of gingival mesenchymal stem cells by inhibiting HMGA2 under nifedipine treatment

**DOI:** 10.1038/s41368-020-0076-8

**Published:** 2020-03-31

**Authors:** Xiao Han, Ruzhuang Yang, Haoqing Yang, Yangyang Cao, Nannan Han, Chen Zhang, Ruitang Shi, Zhengting Zhang, Zhipeng Fan

**Affiliations:** 1 Beijing Key Laboratory of Tooth Regeneration and Function Reconstruction, Beijing Stomatological Hospital, School of Stomatology, Capital Medical University, Beijing, China; 2 Department of Prosthodontics, Beijing Stomatological Hospital, School of Stomatology, Capital Medical University, Beijing, China; 3 Department of Stomatology, First Hospital of Qinhuangdao, Qinhuangdao, China; 4 Department of Periodontology, Beijing Stomatological Hospital, School of Stomatology, Capital Medical University, Beijing, China; 5 Department of Endodontics, Beijing Stomatological Hospital, School of Stomatology, Capital Medical University, Beijing, China

**Keywords:** Molecular biology, Mesenchymal stem cells

## Abstract

Drug-induced gingival overgrowth (DIGO) is recognized as a side effect of nifedipine (NIF); however, the underlying molecular mechanisms remain unknown. In this study, we found that overexpressed miR-4651 inhibits cell proliferation and induces G0/G1-phase arrest in gingival mesenchymal stem cells (GMSCs) with or without NIF treatment. Furthermore, sequential window acquisition of all theoretical mass spectra (SWATH-MS) analysis, bioinformatics analysis, and dual-luciferase report assay results confirmed that high-mobility group AT-hook 2 (HMGA2) is the downstream target gene of miR-4651. Overexpression of HMGA2 enhanced GMSC proliferation and accelerated the cell cycle with or without NIF treatment. The present study demonstrates that miR-4651 inhibits the proliferation of GMSCs and arrests the cell cycle at the G0/G1 phase by upregulating cyclin D and CDK2 while downregulating cyclin E through inhibition of HMGA2 under NIF stimulation. These findings reveal a novel mechanism regulating DIGO progression and suggest the potential of miR-4651 and HMGA2 as therapeutic targets.

## Introduction

Drug-induced gingival overgrowth (DIGO) is a tissue-specific oral disease that involves hyperplasia and hypertrophy of the gingiva. DIGO is an adverse drug reaction related largely to three types of medicines: antiepileptic drugs, immunosuppressants and calcium channel blockers (CCBs).^1–3^ Gingival overgrowth is a major problem in maintaining oral hygiene, increasing the patient’s vulnerability to oral infection, inflammation and periodontal disease. Currently, the treatment for DIGO includes good oral hygiene, periodontal therapy, gum resection and dose control of harmful drugs. However, when it is not possible to remove or replace the drug, postoperative recurrence and treatment of gingivitis are common.^4^ Although gingival overgrowth is not directly life-threatening, the quality of life of affected individuals is impaired. To improve treatment options in the future, the molecular mechanisms of DIGO need to be characterized.

CCBs are a widely used group of antihypertensive drugs. It has been reported that the prevalence of nifedipine-induced gingival overgrowth (NIGO) is 20%–83%, whereas the average compound rate of gingival overgrowth in patients taking other CCBs, such as verapamil, diltiazem, felodipine, or amlodipine, is ~5%.^5,6^ In 2010 and 2011, global sales of generic and non-patented nifedipine tablets for the treatment of hypertension were $1.2 billion.^7^ As the frequency of nifedipine use increases, nifedipine-induced gingival enlargement will continue to increase. NIGO induces cell growth and accumulation of extracellular matrix in the lamina propria connective tissue, leading to epithelial proliferation and elongation.^8^ Recent studies have shown that NIGO is similar to fibrosis, and epithelial–mesenchymal transition (EMT) is involved in NIGO development.^9^ EMT is a process of cell transdifferentiation in which epithelial cells lose contact with each other and acquire characteristics typical of mesenchymal cells.^10^ To better understand this pathological process, it is important to investigate the characteristics and molecular mechanisms of epithelial cells and mesenchymal cells in NIGO.

Recently, gingival mesenchymal stem cells (GMSCs) have been isolated and identified.^11^ GMSCs demonstrate pluripotency with hyperproliferation and the characteristics of MSCs.^12^ Compared with other MSCs, GMSCs are abundant in quantity and easy to obtain by minimally invasive cell-separation technology.^13^ Furthermore, the dynamic physiological and pathophysiological processes of gingival tissue seem to be connected to functional changes in GMSCs. Recently, a study found that inducing GMSCs to differentiate into a pro-fibrotic phenotype in an inflammatory microenvironment may be the basis of inflammatory gingival hyperplasia.^14^ Therefore, we have reason to believe that GMSC dysfunction is closely associated with DIGO.

Epigenetics can explain many phenomena that genetics cannot. For example, recent evidence shows that the ectopic endometrium has a unique epigenetic signature.^15,16^ In addition, DNA methylation patterns, histone modifications and microRNAs (miRNAs) can regulate the proliferation, invasion and apoptosis of endometrial cells.^17^ miRNAs are a type of small, noncoding single-stranded RNA molecules that exert their influence mainly through posttranscriptional processing.^18,19^ Recently, increasing numbers of studies have indicated that miRNAs regulate biological processes such as cell proliferation, apoptosis, the cell cycle and cell differentiation.^20–24^ In addition, miRNAs play an important role in the occurrence, development and prognosis of human cancer.^25^ Previously, our miRNA array analysis results showed that some miRNAs, such as miR-3940-5p and miR-4651, were differentially expressed in gingiva from patients treated with NIF. Our research showed that miR-3940-5p inhibits the proliferation of GMSCs, arresting the cell cycle at the G0/G1 phase. The study indicated that miRNA is a promising target for DIGO therapy.^26^ Therefore, it may be useful to explore the function of other candidate miRNAs to identify more targets and mechanisms. Another previous study suggested that serum miR-4651 may be a useful marker for the diagnosis and prognosis of hepatocellular carcinoma, especially in AFB1-positive cases.^27^ It has recently been reported that miR-4651 regulates nonsense-mediated mRNA decay by inhibiting SMG9 mRNA expression.^28^ However, the role of miR-4651 in DIGO is poorly understood.

In the present study, we examined the function and mechanism of miR-4651 in GMSCs treated with NIF. Our findings revealed that miR-4651 inhibits the proliferation of GMSCs by targeting HMGA2.

## Results

### Overexpression of miR-4651 inhibits cell proliferation and induces G0/G1-phase arrest in GMSCs

**Fig. 1:**
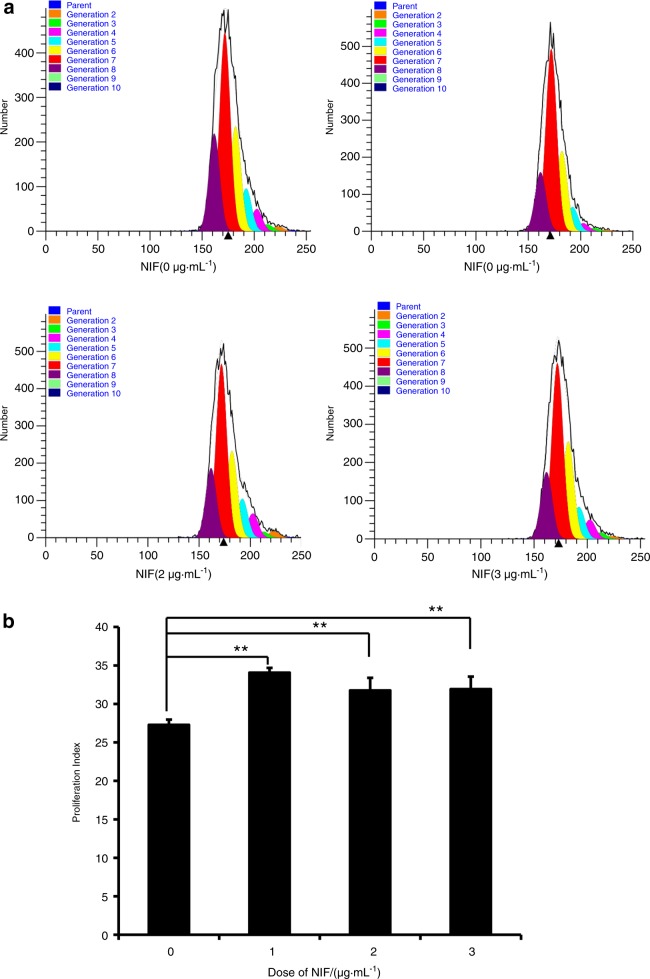
NIF promoted cell proliferation in GMSCs. The CFSE assay indicated that NIF (1, 2, or 3 μg·mL^−1^) promoted cell growth. One-way ANOVA was used to analyse the statistical significance. All error bars indicate standard deviations (*n* = 3). **P ≤ 0.01

**Fig. 2:**
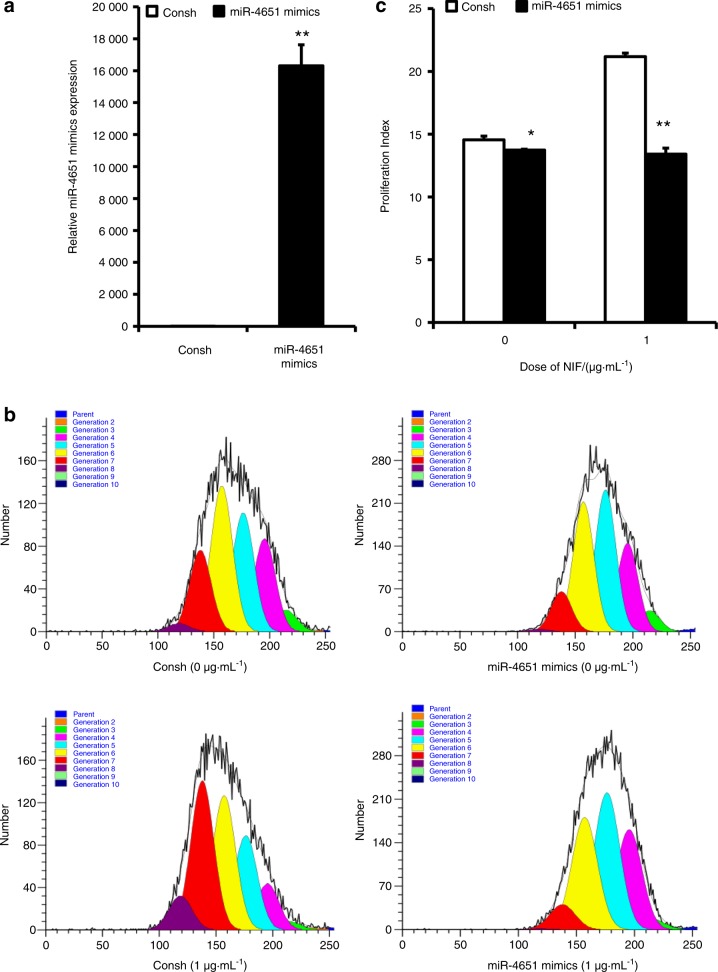
Overexpression of miR-4651 suppressed cell proliferation in GMSCs. **a** miR-4651 overexpression in GMSCs. **b**, **c** CFSE assay revealed that the miR-4651 mimic inhibited cell proliferation in GMSCs treated with 1 μg·mL^−1^ NIF or without NIF. Student’s *t*-test was used to analyse the statistical significance. All error bars indicate standard deviations (*n* = 3). *P ≤ 0.05, **P ≤ 0.01

**Fig. 3:**
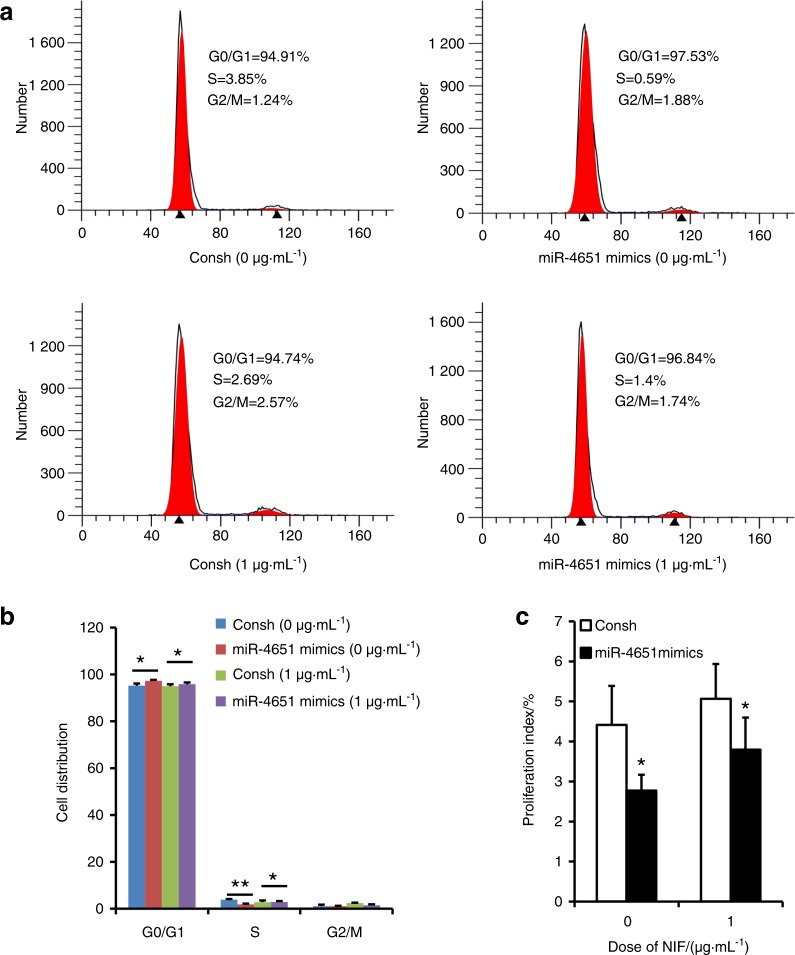
Overexpressed miR-4651 induced G0/G1 arrest in GMSCs. **a**, **b** The flow cytometry analysis results showed an increased cell percentage in the G0/G1 phase and a decreased cell percentage in the S phase in miR-4651-overexpressing GMSCs treated with 1 μg·mL^−1^ NIF or without NIF. **c** The proliferation index was calculated based on flow cytometry results. Student’s *t*-test was used to analyse the statistical significance. All error bars indicate standard deviations (*n* = 3). *P ≤ 0.05, **P ≤ 0.01

We examined MSC markers in GMSCs by flow cytometric analysis. The results showed that the surface markers CD90, CD146 and CD105 were positively expressed in GMSCs (Supplementary Fig. 1). CFSE assay results indicated that growth of GMSCs was promoted by NIF (1, 2 and 3 μg·mL^−1^) compared to cell growth without NIF stimulation; there was no difference in the proliferation of GMSCs among the different doses of NIF (Fig. 1). To investigate the role of miR-4651 in the proliferation of GMSCs, cells were infected with a lentiviral miR-4651 mimic. After selection with 1 µg·mL^−1^ puromycin for 3 days, overexpression of miR-4651 in GMSCs was confirmed by real-time RT-PCR (Fig. 2a). CFSE results showed that the miR-4651 mimic inhibited the growth of GMSCs, even when treated with 1 μg·mL^−1^ NIF (Fig. 2b, c). We then performed a cell cycle assay by flow cytometry to further verify whether miR-4651-mediated inhibition of proliferation was related to cell cycle progression. The number of cells in G0/G1 phase increased significantly, and the number in S phase was reduced in miR-4651-overexpressing GMSCs compared with the control group. This was true with or without 1 μg·mL^−1^ NIF stimulation, and the percentage of cells in G2/M phase was not different, regardless of the treatment group (Fig. 3a, b). Moreover, the proliferation index results revealed that the miR-4651 mimic suppressed the proliferation of GMSCs regardless of treatment with 1 μg·mL^−1^ NIF (Fig. 3c).

### miR-4651 directly inhibits HMGA2 in GMSCs

**Fig. 4:**
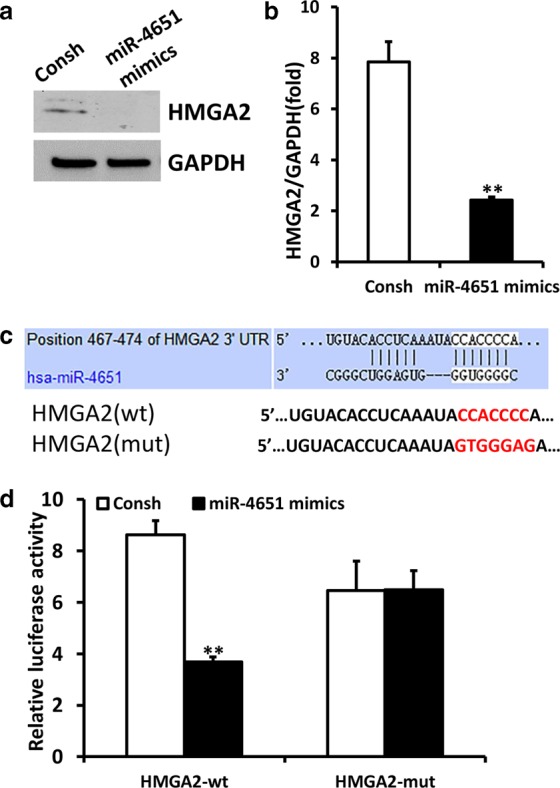
miR-4651 negatively regulated HMGA2 in GMSCs. **a**, **b** GMSCs were transfected with a negative control (Consh) or miR-4651 mimic, and HMGA2 expression was then measured by western blot and real-time RT-PCR. **c** miR-4651 aligned with the 3′UTR of HMGA2. **d** HEK293T cells cotransfected with a reporter carrying a mutant or wild-type (wt) HMGA2 3′UTR and miR-4651 mimic or Consh were analysed by luciferase assay. Data are represented as the mean ± SEM (*n* = 3). **P ≤ 0.01

To clarify the mechanism of miR-4651 in GMSCs, we utilized sequential window acquisition of all theoretical mass spectra (SWATH-MS) to discover downstream proteins of miR-4651. Next, by comparing miR-4651 mimic and control GMSCs, we found 265 proteins significantly differentially expressed between these two groups, of which 136 were upregulated and 129 were downregulated (Supplementary Table 1). Moreover, the TargetScan, miRTarBase and PicTar online tools were used to predict potential targets of miR-4651. Using these combined approaches, we identified HMGA2 as a candidate target gene of miR-4651. Western blot results indicated that the miR-4651 mimic significantly decreased HMGA2 expression at the protein level (Fig. 4a). Furthermore, real-time RT-PCR results indicated that overexpressed miR-4651 significantly repressed HMGA2 expression at the mRNA level (Fig. 4b). Potential binding sites of miR-4651 in the 3ʹUTR of HMGA2 were predicted using TargetScan software (Fig. 4c). Luciferase reporters for the wild-type and mutant HMGA2 3′UTRs were constructed and cotransfected with negative control or miR-4651 mimic into 293T cells. Luciferase assay results revealed that the miR-4651 mimic inhibited the luciferase activity of the wild-type HMGA2 3′UTR reporter but did not inhibit the luciferase activity of the mutant HMGA2 3′UTR reporter (Fig. 4d).

### Overexpression of HMGA2 promotes cell proliferation and cell cycle progression in GMSCs

**Fig. 5:**
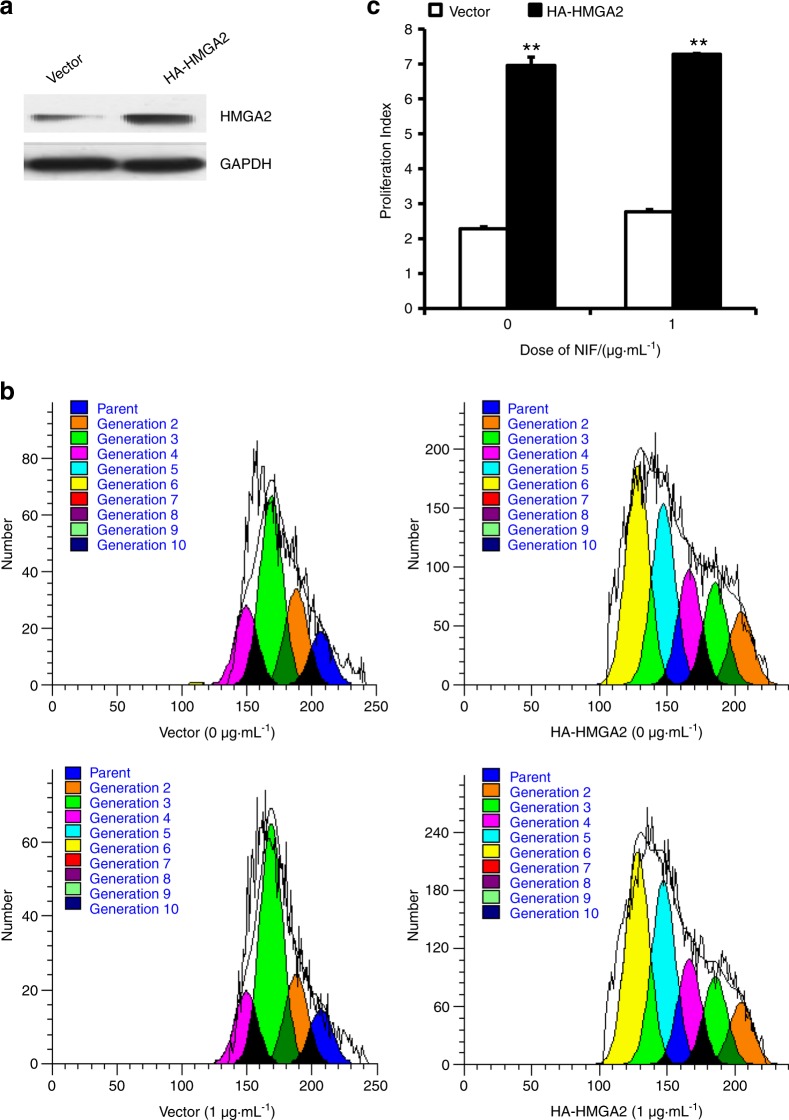
Overexpression of HMGA2 promoted the proliferation in GMSCs. **a** HA-HMGA2 overexpression in GMSCs was measured by western blot. **b**, **c** CFSE assay revealed that HMGA2 overexpression promoted cell proliferation in GMSCs treated with 1 μg·mL^−1^ NIF or without NIF. Student’s *t*-test was used to analyse the statistical significance. All error bars indicate standard deviations (*n* = 3). **P ≤ 0.01

**Fig. 6:**
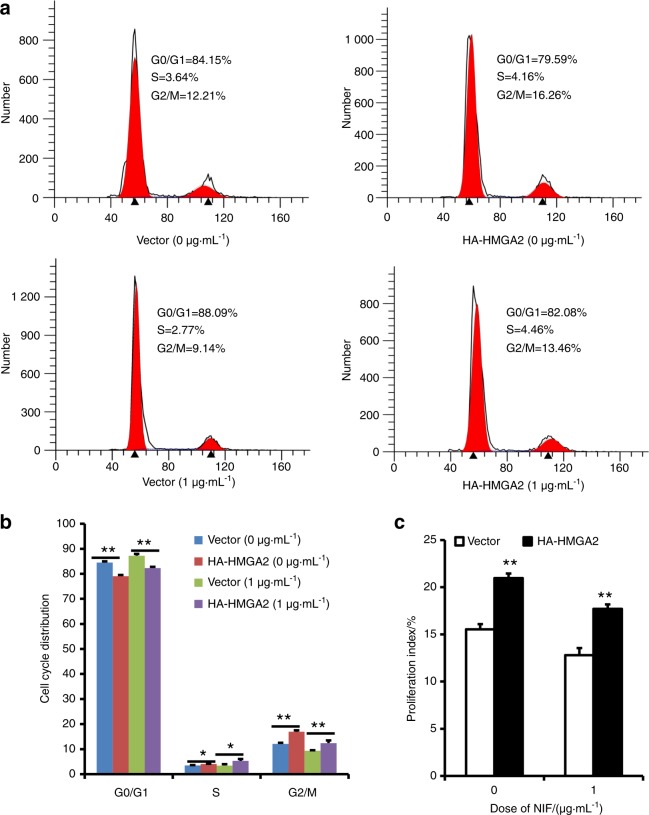
Overexpression of HMGA2 accelerated the cell cycle in GMSCs. **a**, **b** The flow cytometric analysis results showed a decreased cell percentage in the G0/G1 phase and an increased cell percentage in the S and G2/M phases in HMGA2-overexpressing GMSCs treated with 1 μg·mL^−1^ NIF or without NIF. **c** The proliferation index was calculated based on flow cytometry results. Student’s *t*-test was used to analyse the statistical significance. All error bars indicate standard deviations (*n* = 3). *P ≤ 0.05, **P ≤ 0.01

To further investigate the function of HMGA2 in GMSCs, we transfected HA-HMGA2 or empty vector into GMSCs. After selection with 600 µg·mL^−1^ G418 for 10 days, the overexpression efficiency of HMGA2 in GMSCs was detected by western blot (Fig. 5a). A CFSE assay was then conducted to explore the effect of HMGA2 on cell growth. CFSE results showed that HMGA2 overexpression boosted cell proliferation compared with that of the control group, both without treatment and under 1 μg·mL^−1^ NIF stimulation (Fig. 5b, c). Cell cycle results indicated that HMGA2 overexpression decreased the percentage of cells in G0/G1 phase and augmented the percentage in S and G2/M phases in GMSCs compared with the control group, regardless of whether the cells were treated with 1 μg·mL^−1^ NIF (Fig. 6a, b). The proliferation index data also revealed that HMGA2 overexpression promoted the proliferation of GMSCs with or without 1 μg·mL^−1^ NIF treatment (Fig. 6c).

### miR-4651 and HMGA2 regulate the expression of cell cycle-associated proteins in GMSCs under NIF stimulation

**Fig. 7:**
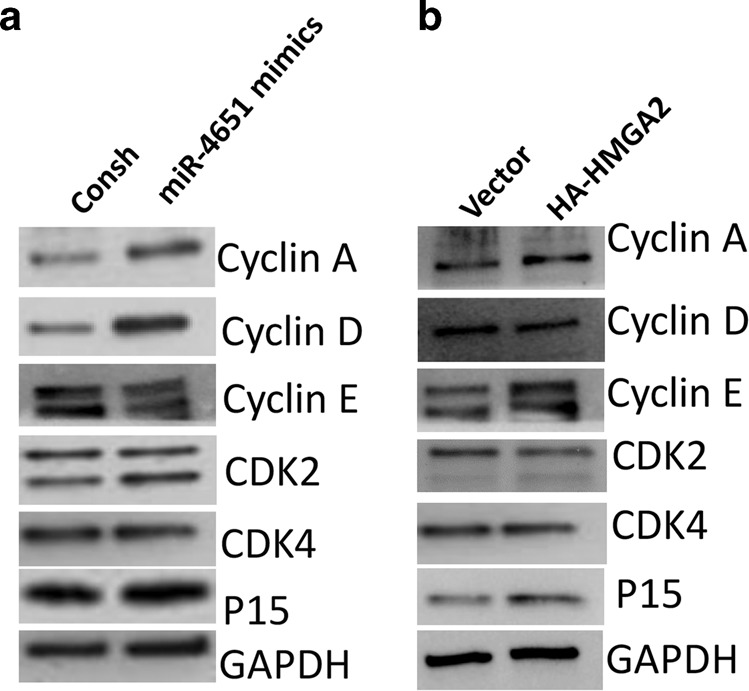
Overexpression of miR-4651 or HMGA2 regulated the expression of Cyclin A, Cyclin D, Cyclin E, CDK2 and p15^INK4b^ at the protein level in GMSCs. **a** Western blot results showing the expression of Cyclin A, Cyclin D, Cyclin E, p15^INK4b^, CDK2 and CDK4 in GMSCs overexpressing miR-4651 and treated with 1 μg·mL^−1^ NIF. **b** The western blot results showed the expression of Cyclin A, Cyclin D, Cyclin E, p15^INK4b^, CDK2 and CDK4 in HMGA2-overexpressing GMSCs treated with 1 μg·mL^−1^ NIF. GAPDH was used as an internal control

Next, to explore the effect of miR-4651 and HMGA2 on the cell cycle, we determined the expression of specific cell cycle regulators at the protein level. Upon overexpression of miR-4651, the protein levels of p15INK4b, cyclin A, cyclin D, and CDK2 increased, while the cyclin E level decreased under treatment with 1 μg·mL^−1^ NIF in comparison with the control group (Fig. 7a). In contrast, the level of CDK4 was not significantly different between miR-4651-overexpressing GMSCs and the control group (Fig. 7a). Furthermore, overexpression of HMGA2 upregulated the protein levels of p15INK4b, cyclin A, and cyclin E and downregulated the levels of cyclin D and CDK2 compared with the control group under treatment with 1 μg·mL^−1^ NIF (Fig. 7b). In contrast, the level of CDK4 was not significantly different between HMGA2-overexpressing GMSCs and the control group (Fig. 7b).

## Discussion

It has been shown that gum overgrowth promotes cell growth and inhibits apoptosis during drug treatment and gingival tissue inflammation.^29^ In this study, we found that 1 μg·mL^−1^, 2 μg·mL^−1^ and 3 μg·mL^−1^ NIF boosted the growth of GMSCs compared to cells untreated with NIF and that there was no difference in the effect among the three doses of NIF. The results indicate that the growth of GMSCs was increased by NIF stimulation. Because the effect was not dose-dependent, 1 μg·mL^−1^ NIF was used in further experiments. We discovered that miR-4651 inhibited GMSC growth with or without 1 μg·mL^−1^ NIF. Subsequently, we detected the effect of miR-4651 on the cell cycle in GMSCs, finding that miR-4651 increased the number of cells in G0/G1 phase and decreased the number of cells in S phase, with or without NIF treatment. These findings suggest that miR-4651 inhibits the growth of GMSCs by blocking cells in G0/G1 phase regardless of NIF treatment and indicate that miR-4651 might play a critical role in DIGO, implicating miR-4651 as a potential therapeutic target for NIGO.

Studies have shown that miRNAs play a role in inhibiting translation or promoting degradation of mRNAs.^30,31^ Increasing evidence indicates that miRNAs directly or indirectly target transcripts encoding proteins associated with cell proliferation and cell cycle progression.^32,33^ Furthermore, our SWATH-MS analyses revealed that several upregulated and downregulated proteins have functions related to cell proliferation or the cell cycle. For example, KCTD12 and FSTL1 were found to be downregulated proteins in SWATH-MS analyses. KCTD12 activates CDK1 and aurora A by binding to CDC25B to facilitate the transition of G2/M phase and promote tumourigenesis.^34^ FSTL1 inhibits the invasion, proliferation, and survival of non-small cell lung cancer tumour cells.^35^ Galectin-8 and BASP1 were identified as upregulated proteins. Galectin-8 acts as a cell-growth regulator by upregulating p21.^36^ Overexpression of BASP1 promotes colony formation, cell proliferation, cell cycle progression, and tumourigenicity, while knocking out BASP1 has the opposite effects.^37^ Then, miRTarBase, TargetScan, and PicTar were used to predict the potential targets of miR-4651, and HMGA2 was identified as a candidate target gene. HMGA2 is a small nuclear protein that belongs to the high-motility group (HMG) protein family, which is generally present in undifferentiated mesenchymal tissues.^38^ HMGA2 includes the basic AT-hook domain that gives it the ability to bind to DNA grooves on sequences rich in A and T nucleotides, as well as the ability to assemble transcriptional or enhancer complexes on chromatin.^39^ Several studies have shown that HMGA2 regulates cell cycle progression, cell differentiation, and cell senescence by enhancing or inhibiting the expression of multiple genes.^40,41^ Many studies have shown that HMGA2 acts as the direct target of different miRNAs, including miR-98, miR-204-3p, and miR-490-3p.^42–44^ In the present study, western blotting and real-time RT-PCR results indicated that HMGA2 was negatively regulated by miR-4651, further supporting the hypothesis that HMGA2 might be the target of miR-4651. In addition, the dual-luciferase reporter assay revealed that miR-4651 directly targeted the binding element of the 3ʹUTR of HMGA2. These data confirmed that HMGA2 may be a direct target gene of miR-4651 in GMSCs. Next, we investigated whether miR-4651 affects cell proliferation and the cell cycle via HMGA2. We found that overexpressed HMGA2 promoted cell proliferation and accelerated cell cycle progression by arresting cells in the S and G2/M phases while decreasing the proportion of cells in the G0/G1 phase, with or without NIF treatment. In conclusion, the present study reveals that the effect of miR-4651 on G0/G1 and S phase in GMSCs produced effects that were opposite to those of HMGA2. The results demonstrated that miR-4651 inhibited the proliferation of GMSCs and blocked the cell cycle at G0/G1 phase, with or without NIF stimulation, by inhibiting HMGA2. miR-4651 had no effect on G2/M phase in GMSCs, with or without treatment with 1 μg·mL^−1^ NIF, while HMGA2 increased the number of GMSCs in G2/M phase. These results suggest that HMGA2 plays a different role in G2/M regulation in GMSCs than miR-4651.

Cells in the body are maintained by cell cycle regulation, which controls cell proliferation.^45^ Cell cycle arrest can be caused by multiple stimulators, which may lead to cell division inhibition, cell death, and/or apoptosis.^46^ Our study revealed that miR-4651 had inhibitory effects on GMSC cell growth by inducing significant G0/G1-phase cell accumulation, whereas HMGA2 promoted GMSC cell proliferation by increasing the number of cells in S and G2/M phases. To further investigate the molecular basis of this observation, we assessed the possible factors involved in cell cycle regulation under NIF treatment by western blot. In cell cycle regulation, the cyclin E/CDK2 complex facilitates cell entry into S phase through the late stage of G1 to regulate the progression of the cell cycle.^47^ Moreover, the cyclin A/CDK2 complex prevents cells from entering the G0/G1-S phase.^48^ In this study, we found that miR-4651 significantly upregulated cyclin A and CDK2 and downregulated cyclin E. Our findings indicate that miR-4651 may induce G0/G1 phase by upregulating cyclin A and CDK2 and downregulating cyclin E. In addition, in the early stage of G1, the formation of cyclin D and CDK4/6 complexes drives entry into the cell cycle.^49^ p15INK4b, a cyclin-dependent kinase inhibitor (CKI), may postpone the progression of the G0/G1 phase by suppressing the activity of the cyclin D/CDK4 or cyclin D/CDK6 complex.^50^ Interestingly, our results revealed that miR-4651 increases the expression levels of p15INK4b and cyclin D. However, CDK4 expression did not vary significantly under our testing conditions. The findings reveal that miR-4651 may also increase the number of cells in G0/G1 phase by upregulating p15INK4b and cyclin D. Moreover, HMGA2 increased cyclin A and cyclin E and decreased CDK2 in GMSCs under NIF stimulation. This indicates that HMGA2 might increase the percentage of cells in S phase by modulating protein levels in GMSCs. In addition, HMGA2 increased the expression levels of p15INK4b and decreased cyclin D, indicating that HMGA2 may inhibit cells in the G0/G1 phase by downregulating cyclin D and upregulating p15INK4b. HMGA2 also increases the percentages of cells in the G2/M phase by regulating CDK1 and cyclin B, which needs further exploration.^51^ Overall, our results suggest that miR-4651 regulates cell proliferation and suppresses the cell cycle at the G0/G1 phase by upregulating CDK2 and cyclin D and downregulating cyclin E through inhibition of HMGA2. However, our results also showed that the roles and mechanism of HMGA2 and miR-4651 in cell cycle regulation have some differences. Several other genes downstream of miR-4651 might also be involved in cell cycle regulation, such as TRIM22, another protein found by our SWATH-MS analysis. It has been reported that TRIM22 downregulation significantly induces cell cycle arrest by regulating the levels of CDK4, cyclin D1, P70S6K and P53 in K562 cells.^52^ In summary, these findings indicate that HMGA2 might be a functional target of miR-4651 in the regulation of cell proliferation in GMSCs under NIF stimulation, and the miR-4651-HMGA2 signalling pathway may be involved in the process of NIGO.

To our knowledge, this is the first study to examine the role of miR-4651 and HMGA2 in regulating proliferation in NIGO. Our results provide a basis for targeting the miR-4651-HMGA2 signalling axis for the prevention and treatment of NIGO. This study further supports our previous studies and shows that miRNA plays an important role in the regulation of drug-induced gingival hyperplasia.^26^ These findings provide a new research direction for the study of drug-induced gingival hyperplasia in the future, as well as a new theoretical basis for research and development of clinical therapeutic drugs in the future. However, this study had some limitations. It has been reported that gingival fibroblasts can cause gingival overgrowth.^53^ The current study focused only on the effects of miR-4651 on GMSCs. Therefore, investigation of gingival fibroblasts is needed to fully elucidate the impact of miR-4651 on NIGO. In the present study, nifedipine was used only to establish cellular NIGO models, and the pathogenesis needs to be further verified in animal experiments.

### Conclusion

The present study indicated that miR-4651 inhibits cell proliferation by arresting cells in the G0/G1 phase via HMGA2 in GMSCs by upregulating cyclin D and CDK2 and downregulating cyclin E. It also explored the role and mechanism of the miR-4651-HMGA2 signalling pathway in regulating cell proliferation and the cell cycle in GMSCs under NIF treatment. Finally, this work has provided a promising therapeutic target for NIGO treatment in the future.

## Materials and methods

### Cell culture

With the approval of the Beijing Stomatology Hospital, School of Stomatology, Capital Medical University (Ethics Review No. 2011-02), gingival tissues were collected from six healthy patients without history of periodontal disease, such as routine dental surgeries. Informed consent was provided by all patients for the use of their tissues. The tissues were treated aseptically and washed with solutions of phosphate-buffered saline (PBS). GMSCs were isolated and cultured as previously described.^11^ Briefly, cells were grown in a humidified atmosphere of 5% CO_2_ at 37 °C, and the medium was changed every 3 days. Cells obtained from generations 3 to 5 were used in subsequent studies.

Drug formulation was performed as previously described.^26^ Briefly, the original solution was diluted to a specified concentration of nifedipine with MSC medium.

### Synthesis of miRNA and plasmid construction

A lentivirus miR-4651 mimic was designed and synthesized by GenePharma (Suzhou, China). The miR-4651 mimic sequence was 5′-CGAAACGGGGTGGGTGA-3′, and the Consh sequence was 5′- TATGGTTGTTCACGACTCCTTCAC-3′. According to standard techniques, we constructed the plasmids and tested all constructs by proper restriction enzymes and/or sequences. For gene synthesis, the human full-length HMGA2 gene sequence was combined with an HA-tag, and then this sequence (HA-HMGA2) was subcloned into the pQCXIN retroviral vector at the BamHI and PacI restriction sites. According to the manufacturer’s protocols, for viral infection, GMSCs were seeded and then infected with retroviruses or lentiviruses in the presence of polybrene (6 mg·mL^−1^, GenePharma) for 12 h. After 48 h, infected cells were selected by using 1 µg·mL^−1^ puromycin for 3 days or 600 µg·mL^−1^ G418 for 10 days.

### Flow cytometry analysis of GMSCs

MSCs at passage 3 were digested by 0.25% trypsin, washed twice with PBS, and then used as a single cell suspension. Approximately 2.0 × 10^5^ cells were incubated with fluorescence-coupled antibodies for 30 min at 4 °C and analysed by flow cytometry (Calibur; BD Biosciences) with FlowJo 10. The antibodies used were as follows: FITC anti-rat CD90 antibody (Cat No. 328108, BioLegend, USA), FITC anti-CD105 antibody (Cat No. 323204, BioLegend, USA) and FITC anti-mouse CD146 antibody (Cat No. 134706, BioLegend, USA).

### CFSE assay

According to the CellTrace CFSE Cell Proliferation Kit protocol (Invitrogen, USA), we performed the CFSE assay. GMSCs were labelled with CFSE in suspension, inoculated in 6-well plates with 1.0 × 10^5^ cells/plate and incubated overnight. The next day, we replaced the medium with 2 mL of MSC medium containing NIF at concentrations of 0, 1, 2 or 3 μg·mL^−1^ and continued to culture the cells for 48 h. Proliferating cells were harvested by 0.25% trypsin and analysed by flow cytometry (Calibur; BD Biosciences). The proliferation index was calculated by MODFIT LT.

### Cell cycle assay

The transfected GMSCs were inoculated in 6-well plates at 1.0 × 10^5^ cells per mL for 24 h and then treated with or without NIF (1 μg·mL^−1^) for 48 h. After culturing for 48 h, the cells were harvested by 0.25% trypsinization. The digested cells were washed with PBS and fixed with 70% alcohol at 4 °C for >24 h. Next, the cells were washed with cold PBS and resuspended in PBS. Following the manufacturer’s instructions, the cells were stained with a propidium iodide (PI) working solution (100 µg·mL^−1^ RNase A and 100 µg·mL^−1^ PI, Sigma) at 37 °C for 30 min away from light. DNA content was evaluated by flow cytometry. MODFIT LT was used to determine the cell cycle distribution. The proliferation index was calculated as PI = (S + G2/M)/(G0/G1 + S + G2/M).

### Dual-luciferase reporter assays

293T cells were inoculated in 6-well plates at 2.0 × 10^5^ cells per mL before transfection. Then, miR-4651 mimic or miR-NC and the wild-type (wt) or mutant (mut) HMGA2 3′UTR-Luc reporter construct were cotransfected into 293T cells by Lipofectamine 3000 (Invitrogen). After 48 h, the cells were harvested, and luciferase enzymatic activities were measured by the Dual-Luciferase Reporter Assay System (E1910; Promega, Madison, WI, USA).

### Reverse transcription-polymerase chain reaction (RT-PCR) and real-time RT-PCR

Total RNA from GMSCs was extracted by using TRIzol (Invitrogen). The levels of miR-4651 in GMSCs were detected by using a Hairpin-it microRNA and U6 snRNA Normalization RT-PCR Quantitation Kit (GenePharma, Suzhou, China). Two-microgram aliquots of RNA were reverse-transcribed into cDNA with random hexamers or oligo(dT) and reverse transcriptase according to the manufacturer’s instructions (Invitrogen). Then, real-time PCR was performed as previously described.^54^ GAPDH and U6 were used to normalize mRNA and miRNA levels, respectively. Primer sequences are listed in Table 1.

**Table 1:** Primers sequences used in the real-time RT-PCR

Gene symbol	Primer sequence (5′-3′)
GAPDH-F	CGGACCAATACGACCAAATCCG
GAPDH-R	AGCCACATCGCTCAGACACC
HMGA2-F	ACCCAGGGGAAGACCCAAA
HMGA2-R	CCTCTTGGCCGTTTTTCTCCA

### Western blot

Total proteins obtained from GMSCs transfected miR-4651 mimic and HA-HMGA2, were resolved, and SDS polyacrylamide gel tests were performed according to a previous protocol.^54^ In this study, the primary antibodies against HMGA2 (Cat No. 5269, Cell Signaling Technology, USA), Cyclin E (Cat No. 05-363, Merck Millipore, Darmstadt, Germany), CDK2 (Cat No. 05-163, Merck Millipore), CDK4 (Cat No. MAB8879, Merck Millipore), Cyclin A (Cat No. SAB4503499, Sigma-Aldrich), Cyclin D (Cat No. 05-152, Merck Millipore) and p15INK4B (Cat No. 4822, Cell Signaling Technology, USA) were used, and an antibody glyceraldehyde 3-phosphate dehydrogenase (GAPDH; Cat No. G8795, Sigma-Aldrich) was used as a housekeeping protein

### Statistical analysis

Statistical calculations were implemented using SPSS 10 statistical software. Statistical significance was analysed by one-way ANOVA or Student’s *t*-test; *P* ≤ 0.05 was considered significant.

## Supplementary information

Supplementary Table 1

Supplementary Figure 1

Supplementary Figure Legend

## Data Availability

Research data are not shared.
