# Supplementary material for: miR-4651 inhibits cell proliferation of gingival mesenchymal stem cells by inhibiting HMGA2 under nifedipine treatment
Source: Int J Oral Sci. 2020 Mar 31;12:10. doi: 10.1038/s41368-020-0076-8 (PMC7105500; doi:10.1038/s41368-020-0076-8)
Supplement: Supplementary file 1 — Supplementary Table 1 [file 41368_2020_76_MOESM1_ESM.pdf]

Supplementary Table 1. Sequential window acquisition of all theoretical mass spectra in miR-4651-overexpressing GMSCs.

Peak Name	Protein symbols	Fold Change=miR-4651 mimic/Consh
sp|P53803|RPAB4_HUMAN	POLR2K	0.15421669
sp|Q96SK2|TM209_HUMAN	TMEM209	0.21525916
tr|H0Y6V6|H0Y6V6_HUMAN	LMBR1	0.24133906
tr|M0QZ22|M0QZ22_HUMAN	SAMD4B	0.24282364
sp|P49590|SYHM_HUMAN	HARS2	0.35460121
sp|Q8N8N7|PTGR2_HUMAN	PTGR2	0.38422261
sp|Q8IWR0|Z3H7A_HUMAN	ZC3H7A	0.38634046
tr|D6RF48|D6RF48_HUMAN	STX18	0.38716401
tr|B8ZZQ6|B8ZZQ6_HUMAN	PTMA	0.38765025
sp|Q00059|TFAM_HUMAN	TFAM	0.38858894
sp|P08243|ASNS_HUMAN	ASNS	0.39673298
tr|F8VQZ7|F8VQZ7_HUMAN	METAP2	0.3974487
sp|Q8IZL8|PELP1_HUMAN	PELP1	0.3987758
sp|Q8NBI6|XXLT1_HUMAN	XXYLT1	0.41527625
tr|F5H2A4|F5H2A4_HUMAN	HMGA2	0.41667341
sp|Q9BU76-2|MMTA2_HUMAN	MMTAG2	0.41989519
sp|P04066|FUCO_HUMAN	FUCA1	0.42151692
sp|Q96E52|OMA1_HUMAN	OMA1	0.44606798
tr|H0YL70|H0YL70_HUMAN	TLE3	0.45036695
sp|Q99504|EYA3_HUMAN	EYA3	0.45236369
sp|Q9Y6M7-6|S4A7_HUMAN	SLC4A7	0.45494484
tr|C9JJN9|C9JJN9_HUMAN	VPS8	0.46355313
sp|Q9Y617|SERC_HUMAN	PSAT1	0.46893241
sp|Q9UQ90|SPG7_HUMAN	SPG7	0.47839823
sp|Q9NXZ2|DDX43_HUMAN	DDX43	0.47956299
tr|F6X2W2|F6X2W2_HUMAN	NEGR1	0.48323382
sp|A0JNW5|UH1BL_HUMAN	UHRF1BP1L	0.48795484
sp|Q7LGA3|HS2ST_HUMAN	HS2ST1	0.49057062
sp|P31944|CASPE_HUMAN	CASP14	0.50271513
sp|O14874|BCKD_HUMAN	BCKDK	0.51168728
sp|Q6PKC3|TXD11_HUMAN	TXNDC11	0.51701022
sp|O60934|NBN_HUMAN	NBN	0.52543522
sp|Q6DHV7|ADAL_HUMAN	ADAL	0.53286461
sp|P61956|SUMO2_HUMAN	SUMO2	0.53293753
sp|Q53GT1|KLH22_HUMAN	KLHL22	0.53450117
sp|P23434|GCSH_HUMAN	GCSH	0.54176166
sp|P29279|CTGF_HUMAN	CTGF	0.54672207
sp|P21980|TGM2_HUMAN	TGM2	0.54842147
sp|O76061|STC2_HUMAN	STC2	0.54997068
tr|Q6GPG7|Q6GPG7_HUMAN	LPAR1	0.5526852
sp|Q01650|LAT1_HUMAN	SLC7A5	0.55353016
sp|Q13257|MD2L1_HUMAN	MAD2L1	0.5544076
sp|P35251|RFC1_HUMAN	RFC1	0.55804017
sp|P54802|ANAG_HUMAN	NAGLU	0.55816687
tr|H0Y9X1|H0Y9X1_HUMAN	TMA16	0.56391195
tr|C9JVN9|C9JVN9_HUMAN	L2HGDH	0.56418062
sp|Q9Y2E4|DIP2C_HUMAN	DIP2C	0.56473908
sp|Q14676|MDC1_HUMAN	MDC1	0.56597982
tr|G5E9A6|G5E9A6_HUMAN	USP11	0.57371072
sp|O95159|ZFPL1_HUMAN	ZFPL1	0.57503845
sp|Q16822|PCKGM_HUMAN	PCK2	0.57612609
tr|J3KQ72|J3KQ72_HUMAN	FBXO6	0.5770694
sp|Q9NV56|MRGBP_HUMAN	MRGBP	0.57883229
sp|Q9BYC5|FUT8_HUMAN	FUT8	0.58255918
tr|X6R700|X6R700_HUMAN	CHTOP	0.58326535
sp|Q96A72|MGN2_HUMAN	MAGOHB	0.58400758

sp|Q02388|CO7A1_HUMAN	COL7A1	0.58703763
sp|O75475|PSIP1_HUMAN	PSIP1	0.5873392
sp|P49748-3|ACADV_HUMAN	ACADVL	0.58772543
tr|F8W689|F8W689_HUMAN	RFX5	0.58824717
tr|A0A087WTA8|A0A087WTA8_H	COL1A2	0.58887954
sp|Q9P287|BCCIP_HUMAN	BCCIP	0.5900259
sp|Q96H79|ZCCHL_HUMAN	ZC3HAV1L	0.59019807
sp|Q9H2P0|ADNP_HUMAN	ADNP	0.59209379
sp|P07093|GDN_HUMAN	SERPINE2	0.59686918
sp|Q969Z0|TBRG4_HUMAN	TBRG4	0.59952359
sp|Q96CX2|KCD12_HUMAN	KCTD12	0.59959073
sp|P49321|NASP_HUMAN	NASP	0.60116215
sp|Q6UN15|FIP1_HUMAN	FIP1L1	0.60152095
tr|A0A0A0MTR1|A0A0A0MTR1_H	CDH13	0.60237607
sp|P26006|ITA3_HUMAN	ITGA3	0.60577943
tr|E7ET40|E7ET40_HUMAN	PLAU	0.60648552
tr|E7EV46|E7EV46_HUMAN	ERCC6-PGBD3	0.60892489
sp|P11387|TOP1_HUMAN	TOP1	0.60990422
sp|Q9P258|RCC2_HUMAN	RCC2	0.61075484
sp|O43175|SERA_HUMAN	PHGDH	0.61146155
sp|Q9BRJ6|CG050_HUMAN	C7orf50	0.61453299
sp|P26358|DNMT1_HUMAN	DNMT1	0.61529208
sp|P50748|KNTC1_HUMAN	KNTC1	0.61553525
sp|O43715|TRIA1_HUMAN	TRIAP1	0.61717127
sp|Q8WVQ1|CANT1_HUMAN	CANT1	0.61843981
sp|Q5JSZ5|PRC2B_HUMAN	PRRC2B	0.62006649
sp|P16401|H15_HUMAN	HIST1H1B	0.62045648
tr|D3DQH8|D3DQH8_HUMAN	SPARC	0.62120713
sp|O95407|TNF6B_HUMAN	TNFRSF6B	0.62190868
sp|P00374|DYR_HUMAN	DHFR	0.62206354
tr|A0A087X0M4|A0A087X0M4_H	SLC4A1AP	0.62323976
sp|Q9BZE4|NOG1_HUMAN	GTPBP4	0.62431373
sp|P11169|GTR3_HUMAN	SLC2A3	0.6255659
sp|Q92688|AN32B_HUMAN	ANP32B	0.62624051
sp|O14495|LPP3_HUMAN	PPAP2B	0.62775624
sp|P16403|H12_HUMAN	HIST1H1C	0.62867184
tr|B1AKV3|B1AKV3_HUMAN	UQCC1	0.62932103
sp|Q12841|FSTL1_HUMAN	FSTL1	0.63071429
sp|Q93084-5|AT2A3_HUMAN	ATP2A3	0.63093262
tr|E9PB61|E9PB61_HUMAN	ALYREF	0.63204685
sp|Q9UHE8|STEA1_HUMAN	STEAP1	0.63250693
sp|Q9NQS3|PVRL3_HUMAN	PVRL3	0.63343508
sp|Q9NR30|DDX21_HUMAN	DDX21	0.63479741
sp|P19338|NUCL_HUMAN	NCL	0.63563097
sp|P13726|TF_HUMAN	F3	0.63631528
sp|Q6ZRP7|QSOX2_HUMAN	QSOX2	0.63800395
sp|Q13614-2|MTMR2_HUMAN	MTMR2	0.63937564
sp|Q9NUU7|DD19A_HUMAN	DDX19A	0.63978635
sp|Q96AY3|FKB10_HUMAN	FKBP10	0.64034271
sp|Q9GZU8|F192A_HUMAN	FAM192A	0.64283493
sp|Q8NAV1|PR38A_HUMAN	PRPF38A	0.64382085
sp|Q9NYL4|FKB11_HUMAN	FKBP11	0.64410216
tr|A0A0A0MRM9|A0A0A0MRM9_	NOLC1	0.64413573
sp|O00541|PESC_HUMAN	PES1	0.64459441
tr|J3KTA4|J3KTA4_HUMAN	DDX5	0.64703447
sp|P43353|AL3B1_HUMAN	ALDH3B1	0.64798434
sp|O60828|PQBP1_HUMAN	PQBP1	0.64864641
sp|Q7Z3C6|ATG9A_HUMAN	ATG9A	0.64867351

sp|Q14103|HNRPD_HUMAN	HNRNPD	0.64963353
sp|Q96K37|S35E1_HUMAN	SLC35E1	0.65439733
sp|Q9H0L4|CSTFT_HUMAN	CSTF2T	0.6560692
sp|Q8IWC1-2|MA7D3_HUMAN	MAP7D3	0.65608619
tr|U3KQC1|U3KQC1_HUMAN	WDR18	0.65660855
sp|P62851|RS25_HUMAN	RPS25	0.6598
sp|P38919|IF4A3_HUMAN	EIF4A3	0.66080922
sp|Q5XUX1-3|FBXW9_HUMAN	FBXW9	0.66149464
sp|Q9BSE5|SPEB_HUMAN	AGMAT	0.66284315
sp|Q14966-3|ZN638_HUMAN	ZNF638	0.66319783
sp|Q9NS00|C1GLT_HUMAN	C1GALT1	0.66348108
sp|Q13435|SF3B2_HUMAN	SF3B2	0.66442595
sp|O15355|PPM1G_HUMAN	PPM1G	0.66470814
sp|Q9BSR8|YIPF4_HUMAN	YIPF4	0.66519508
sp|Q8IYM9|TRI22_HUMAN	TRIM22	0.66570606
sp|P09619|PGFRB_HUMAN	PDGFRB	1.50282696
tr|G3V5N8|G3V5N8_HUMAN	ZFYVE1	1.5040222
sp|Q14697|GANAB_HUMAN	GANAB	1.51496382
sp|P80723|BASP1_HUMAN	BASP1	1.51852169
sp|Q9H792|PEAK1_HUMAN	PEAK1	1.51996436
sp|P35580|MYH10_HUMAN	MYH10	1.52042811
sp|O95210|STBD1_HUMAN	STBD1	1.52270377
sp|O00214|LEG8_HUMAN	LGALS8	1.52359101
tr|J3KQL8|J3KQL8_HUMAN	APOL2	1.53995009
sp|P20810|ICAL_HUMAN	CAST	1.54052382
tr|C9JAX1|C9JAX1_HUMAN	FXN	1.54195849
tr|A0A087WSY9|A0A087WSY9_H	TXNRD1	1.54321659
sp|P35754|GLRX1_HUMAN	GLRX	1.54330164
sp|P21266|GSTM3_HUMAN	GSTM3	1.54487249
tr|C9JF58|C9JF58_HUMAN	OSCP1	1.54571037
sp|P23634|AT2B4_HUMAN	ATP2B4	1.54781457
sp|Q6UVK1|CSPG4_HUMAN	CSPG4	1.55126302
sp|P24844|MYL9_HUMAN	MYL9	1.55460985
sp|Q14644|RASA3_HUMAN	RASA3	1.55931865
sp|Q96KS9|F167A_HUMAN	FAM167A	1.55992896
sp|Q9BXF6|RFIP5_HUMAN	RAB11FIP5	1.56281245
sp|Q8NBP0|TTC13_HUMAN	TTC13	1.56845663
sp|P07858|CATB_HUMAN	CTSB	1.56874924
sp|P16035|TIMP2_HUMAN	TIMP2	1.56905279
sp|A6NFQ2|F115C_HUMAN	FAM115C	1.56963488
sp|Q9BXJ9|NAA15_HUMAN	NAA15	1.5698217
sp|P13716|HEM2_HUMAN	ALAD	1.57084586
sp|Q96JJ7|TMX3_HUMAN	TMX3	1.57553396
sp|P13647|K2C5_HUMAN	KRT5	1.57653658
sp|Q9Y2D0|CAH5B_HUMAN	CA5B	1.57775279
sp|O95810|SDPR_HUMAN	SDPR	1.57801458
sp|P02792|FRIL_HUMAN	FTL	1.59149339
sp|Q6R327|RICTR_HUMAN	RICTOR	1.59314053
sp|Q14571|ITPR2_HUMAN	ITPR2	1.5999854
sp|Q12765|SCRN1_HUMAN	SCRN1	1.60277487
sp|Q99538|LGMN_HUMAN	LGMN	1.60589886
sp|P52732|KIF11_HUMAN	KIF11	1.60802017
sp|Q9NZ43|USE1_HUMAN	USE1	1.61187792
sp|Q96EM0|T3HPD_HUMAN	L3HYPDH	1.61444255
tr|A0A087X0S5|A0A087X0S5_HU	COL6A1	1.61504406
sp|P02545-2|LMNA_HUMAN	LMNA	1.61579148
sp|Q6PI78|TMM65_HUMAN	TMEM65	1.62218592
sp|Q8IYB7|DI3L2_HUMAN	DIS3L2	1.62380361

tr|U3KQP1|U3KQP1_HUMAN	ASNSD1	1.62474087
sp|P54725|RD23A_HUMAN	RAD23A	1.63157895
sp|Q15714|T22D1_HUMAN	TSC22D1	1.63523631
sp|P36955|PEDF_HUMAN	SERPINF1	1.63535846
sp|P14621|ACYP2_HUMAN	ACYP2	1.63629653
sp|Q8N1W1|ARG28_HUMAN	ARHGEF28	1.63631473
sp|O94919|ENDD1_HUMAN	ENDOD1	1.63701276
tr|E7EWV1|E7EWV1_HUMAN	PIGG	1.65321934
sp|Q12923|PTN13_HUMAN	PTPN13	1.66531326
sp|Q9H074|PAIP1_HUMAN	PAIP1	1.66539899
sp|Q8NHH9-4|ATLA2_HUMAN	ATL2	1.66604947
sp|Q9H492|MLP3A_HUMAN	MAP1LC3A	1.67498896
sp|Q04771|ACVR1_HUMAN	ACVR1	1.67645172
tr|D3YTB5|D3YTB5_HUMAN	IRAK1	1.67736885
tr|E9PL10|E9PL10_HUMAN	BTF3L4	1.67873608
sp|Q9Y4I1|MYO5A_HUMAN	MYO5A	1.69265913
sp|Q9BUK6|MSTO1_HUMAN	MSTO1	1.70220644
sp|Q96HE7|ERO1A_HUMAN	ERO1L	1.70440971
sp|Q92783|STAM1_HUMAN	STAM	1.7092478
sp|Q13523|PRP4B_HUMAN	PRPF4B	1.71262239
sp|Q14289|FAK2_HUMAN	PTK2B	1.72337581
tr|D6RE79|D6RE79_HUMAN	MFSD10	1.72490925
sp|Q9BS40|LXN_HUMAN	LXN	1.7283031
sp|Q13164|MK07_HUMAN	MAPK7	1.74173595
sp|Q9Y6Q2-2|STON1_HUMAN	STON1	1.74371627
sp|Q6ZXV5|TMTC3_HUMAN	TMTC3	1.74725563
sp|Q02880|TOP2B_HUMAN	TOP2B	1.74787312
sp|P29373|RABP2_HUMAN	CRABP2	1.75204642
sp|Q04656-2|ATP7A_HUMAN	ATP7A	1.75640092
sp|P63241|IF5A1_HUMAN	EIF5A	1.7607991
P34955	Alpha-1	1.76139411
tr|K7EIE8|K7EIE8_HUMAN	MBD3	1.76275059
sp|O14494|LPP1_HUMAN	PPAP2A	1.76450627
sp|Q92478|CLC2B_HUMAN	CLEC2B	1.77277465
sp|Q8IUD6|RN135_HUMAN	RNF135	1.80297147
sp|P46934-4|NEDD4_HUMAN	NEDD4	1.80467839
sp|P07099|HYEP_HUMAN	EPHX1	1.80657695
sp|P00750|TPA_HUMAN	PLAT	1.82465123
sp|Q04828|AK1C1_HUMAN	AKR1C1	1.82866019
sp|P15104|GLNA_HUMAN	GLUL	1.83029796
sp|Q9NSY1|BMP2K_HUMAN	BMP2K	1.8653335
sp|Q9Y2Y0|AR2BP_HUMAN	ARL2BP	1.87689925
tr|E9PR44|E9PR44_HUMAN	CRYAB	1.88762449
sp|P14618|KPYM_HUMAN	PKM	1.89168785
sp|Q8WX93|PALLD_HUMAN	PALLD	1.89752123
sp|Q9P1F3|ABRAL_HUMAN	ABRACL	1.90843217
sp|Q0ZGT2-2|NEXN_HUMAN	NEXN	1.91183121
sp|Q9BRR6|ADPGK_HUMAN	ADPGK	1.98767714
sp|Q9NVZ3|NECP2_HUMAN	NECAP2	1.99264678
tr|G3V529|G3V529_HUMAN	DDX24	2.00415429
sp|O14733-2|MP2K7_HUMAN	MAP2K7	2.02535747
sp|O00165|HAX1_HUMAN	HAX1	2.050402
sp|P61225|RAP2B_HUMAN	RAP2B	2.0515274
sp|Q99685|MGLL_HUMAN	MGLL	2.05975505
sp|O95873|CF047_HUMAN	C6orf47	2.07860976
tr|S4R371|S4R371_HUMAN	FABP3	2.11073065
sp|Q9NX38|F206A_HUMAN	FAM206A	2.12349648
tr|H0YI09|H0YI09_HUMAN	METTL7A	2.1337307

RRRRRsp|P35968|VGFR2_HUMAN	KDR	2.20341941
sp|P63261|ACTG_HUMAN	ACTG1	2.20761269
tr|K7EIY6|K7EIY6_HUMAN	RNF126	2.23101598
sp|P32455|GBP1_HUMAN	GBP1	2.23241796
sp|Q8TDM0|BCAS4_HUMAN	BCAS4	2.26250619
sp|Q8N556|AFAP1_HUMAN	AFAP1	2.27354916
sp|Q68DK2|ZFY26_HUMAN	ZFYVE26	2.28963233
sp|P01112|RASH_HUMAN	HRAS	2.33097095
sp|P15848|ARSB_HUMAN	ARSB	2.33537088
sp|Q9H2F3|3BHS7_HUMAN	HSD3B7	2.39386963
sp|Q6GYQ0-2|RGPA1_HUMAN	RALGAPA1	2.40664803
sp|P51688|SPHM_HUMAN	SGSH	2.46584069
sp|P12110|CO6A2_HUMAN	COL6A2	2.48539478
sp|Q9Y673|ALG5_HUMAN	ALG5	2.54300367
sp|Q9Y2X3|NOP58_HUMAN	NOP58	2.55991017
sp|Q9H3Z4|DNJC5_HUMAN	DNAJC5	2.56409019
sp|O43504|LTOR5_HUMAN	LAMTOR5	2.60124161
sp|Q01995|TAGL_HUMAN	TAGLN	2.63852269
sp|Q92629|SGCD_HUMAN	SGCD	2.64197468
tr|H7C1F9|H7C1F9_HUMAN	RALGAPA2	2.70472888
tr|C9JF17|C9JF17_HUMAN	APOD	2.71709286
tr|E9PPW7|E9PPW7_HUMAN	NDUFS8	2.8147317
tr|H3BP13|H3BP13_HUMAN	TRAPPC2L	2.82269948
sp|P13645|K1C10_HUMAN	KRT10	2.87468179
sp|Q8WUM0|NU133_HUMAN	NUP133	2.89952079
sp|P35908|K22E_HUMAN	KRT2	2.91959045
sp|Q08AE8|SPIR1_HUMAN	SPIRE1	2.97415518
sp|Q69YL0|NCAS2_HUMAN	NCBP2-AS2	3.06096295
sp|P12273|PIP_HUMAN	PIP	3.12643498
sp|P00325|ADH1B_HUMAN	ADH1B	3.67083842
sp|P04179|SODM_HUMAN	SOD2	3.77427473
P35527	KRT9	3.83541089
sp|P04264|K2C1_HUMAN	KRT1	3.86304738
sp|Q16762|THTR_HUMAN	TST	6.0453586
sp|Q15493|RGN_HUMAN	RGN	8.98412569
