# Supplementary figures and images for: miR-4651 inhibits cell proliferation of gingival mesenchymal stem cells by inhibiting HMGA2 under nifedipine treatment
Source: Int J Oral Sci. 2020 Mar 31;12:10. doi: 10.1038/s41368-020-0076-8 (PMC7105500; doi:10.1038/s41368-020-0076-8)

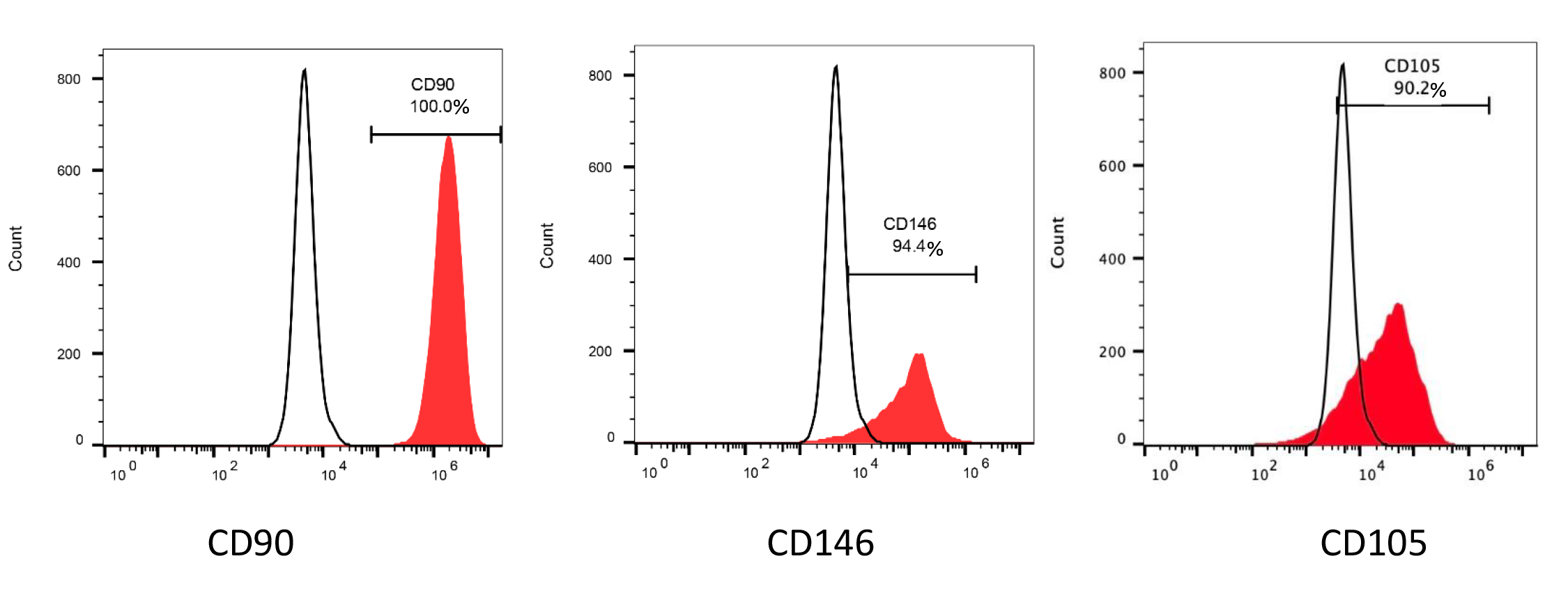

Supplement: Supplementary file 2 — Supplementary Figure 1 [file 41368_2020_76_MOESM2_ESM.tif]
