# Supplementary material for: miR-4651 inhibits cell proliferation of gingival mesenchymal stem cells by inhibiting HMGA2 under nifedipine treatment
Source: Int J Oral Sci. 2020 Mar 31;12:10. doi: 10.1038/s41368-020-0076-8 (PMC7105500; doi:10.1038/s41368-020-0076-8)
Supplement: Supplementary file 3 — Supplementary Figure Legend [file 41368_2020_76_MOESM3_ESM.doc]

**Supplementary Fig. 1. The flow cytometric analysis results showed that CD90, CD146 and CD105 were positively expressed in GMS**
